# Comparison of Demographic and Clinicopathological Characteristics of Basal Cell Carcinoma on the Nose and Other Sites of the Face: A Cross-Sectional Study

**DOI:** 10.22038/ijorl.2021.47720.2575

**Published:** 2021-09

**Authors:** Hossein Kavoussi, Ali Ebrahimi, Mansour Rezaei, Fariba Najafi, Mahsa Zarpoosh, Reza Kavoussi

**Affiliations:** 1 *Department of Dermatology, Hajdaie Dermatology Clinic, School of Medicine, Kermanshah University of Medical Sciences, Kermanshah, Iran.*; 2 *Family Health Research Center, Kermanshah University of Medical Sciences, Kermanshah, Iran.*; 3 *Medical Student, School of Medicine, Students Research Committee, Kermanshah University of Medical Sciences, Kermanshah, Iran.*; 4 *Physical Practitioner, Kermanshah University of Medical Sciences (KUMS), Kermanshah, Iran.*

**Keywords:** Basal cell carcinoma, Face, Nose, Perineural invasion

## Abstract

**Introduction:**

The clinicopathological characteristics of basal cell carcinoma (BCC) in different areas of the face, including the nose, are important and may be different. Accurate recognition of these characteristics may be necessary for the planning and selection of appropriate treatment.

**Materials and Methods:**

This cross-sectional study was conducted on 328 patients (131 females and 197 males) with 371 documented facial BCC in the West of Iran within 2013-2018. The demographic and clinicopathological data of the patients in the nose area were compared with other sites of the face by appropriate statistical methods.

**Results:**

Out of 371 lesions, 38.8% of the cases were on the nose, 75.8% were primary lesions, 97.8% had no perineural invasion, 89.2% were nodular, and 65.8% were of nodular clinical and pathologic type, which were the most common variables of patients. It was revealed that early-onset (P<0.001), smaller size (P<0.001), high-risk pathologic type (P=0.01), and recurrent lesions (P=0.013) were significantly higher in the nasal BCC. However, there was no significant difference between BCC in the nose and other sites of the face in terms of gender (P=0.654), high-risk clinical type (P=0.06), and perineural invasion (P=0.275).

**Conclusion:**

Considering the nasal site as an important cosmetic unit, more limitation of the nose in performing any procedure, and presence of the more risk factors in the nose than in other areas of the face, the definite treatment of nasal BCC requires special attention, expertise, and experience.

## Introduction

Basal cell carcinoma (BCC) is the most common malignancy in human with several clinical and pathologic subtypes. However, the prevalence of BCC in different sites of the face, including the nose, is dependent on racial factors, lifestyle, geographic location, and environmental factors (1-4). The detection of clinicopathological characteristics of BCC lesions is incredibly important in determining the risk factors and their multiplicity, which is highly essential for the selection of the preferred treatment (4-8).

The nose has an important role in respiratory ventilation and cosmetic appearance. The results of numerous studies have reported the nose as the most common site and post-surgical recurrence of BCC. Therefore, any procedure performed in this area for eradication of this malignancy should preserve the integrity of its structure (9,10). Various clinical and pathological variants of BCC that affect its prognosis are highly important in the selection of appropriate optional treatments, especially in excisional biopsy situations. Moreover, some studies have reported high prevalence and different characteristics of nasal BCC than for other sites of the face (OSOF) (4,5,7,11). Consequently, this study aimed to specifically and precisely compare the clinicopathological characteristics of BCC on the nose and OSOF. 

## Materials and Methods

This analytical cross-sectional study was conducted on 328 patients with 371 BCC lesions on the face in a dermatology referral clinic, West of Iran, over 5 years (2013-2018). This study was approved by the Ethical Committee of Kermanshah University of Medical Sciences, Kermanshah, Iran (P/35215/420/7-14/11/ 2014). The participants were informed of the confidentiality in this study. In our study, the samples were selected among the patients who presented to our referral dermatology clinic or patients who were available at the clinic. After recruiting the patients and considering the exclusion criteria, all variables were obtained from all patients; therefore, there no missing data in this study. 

Patients who were clinically suspected of and histopathologically documented with BCC were included in the study. On the other hand, the cases with immunodeficiency, syndromes prone to BCC, unreliable in providing past medical history, and psychological problems were excluded from the study. The required data were collected through interviewing patients or their families by a medical student, performing physical examination by two dermatologists, and conducting pathologic evaluation by a dermatopathologist. The morphoeic, infiltrative, micronodular, superficial, and metatypical forms were considered high-risk pathologic types. Furthermore, morphoeic and superficial clinical types were regarded as high-risk clinical variants (7,8). After obtaining informed written consent from the participants, their demographic data and the previous history, duration, size, location, recurrence, perineural invasion (PNI), and clinicopathological type of BCC were recorded in a questionnaire. In histopathologic assessment, in addition to the diagnosis of BCC and histopathologic type, the PNI was sought, especially in recurrent lesions. In rare cases, when clinical and histopathological evaluation could not definitively differentiate BCC from trichoepithelioma as benign neoplasm, immunohistochemistry was used. The PNI was diagnosed after the total excision of the tumor with an appropriate safe margin. In this situation, multiple sections were prepared using the horizontal technique that permitted the histopathologic examination of all peripheral and deep margins of the resected tumor. 

The collected data were entered into and analyzed in the MS Excel (version 2016) and SPSS software (version 16). Kolmogorov-Smirnov non-parametric test was used to check the normal distribution of quantitative variables, such as age, size, and duration. An independent sample t-test was employed to compare the nasal and OSF groups regarding the variables with normal distribution, such as duration. Mann-Whitney U test was utilized to compare the nasal and OSF groups with respect to the variables without normal distribution, including age and size. Cross tabulation (by number and percent) and Chi-square test were used to compare the nasal and OSF groups in terms of qualitative variables, such as gender. Error bar (with 95% confidence level), Box plot, and Bar chart were used to present the qualitative and normal and non-normal quantitative variables by graphs, respectively. The significance level was considered to be 0.05 for the test analysis. 

## Results

A total of 328 patients (131 females and 197 males) with 371 BCC lesions were recruited in this study, whose mean scores of age, BCC size, and BCC duration were calculated at 56.31±8.871 years, 14.68±7.280 mm, and 41.84± 9.689 months.

It was revealed that the number of lesions ranged from 1 to 6, and 305 (93.0%) subjects had one lesion (Table.1). 

**Table 1:** Demographic and clinicopathological characteristics of patients and basal cell carcinoma lesions

Variables		
**Gender n (%)**	Female Male	131 (39.9) 197 (60.1)
**Mean age**	Years	56.32±8.871
**Mean size**	mm	14.68±7.280
**Mean duration **	Months	41.84±9.689
**Personal history of basal cell carcinoma n (%)**	Yes No	106 (32.3) 222 (67.7)
**Number of lesions n (%)**	One Multiple	305 (93.0) 23 (7.0)
**Location n (%)**	Nose Periorbital Forehead Cheek Periauricular Ear Chin Lateral face Perioral Nasolabial fold Temple	144 (38.8) 57 (15.4) 30 (8.1) 28 (7.5) 21 (5.7) 18 (4.9) 17 (4.6) 17 (4.6) 15 (4.0) 15 (4.0) 9 (2.4)
**Primary or recurrence lesion n (%)**	Primary Recurrence	281 (75.8) 90 (24.2)
**Clinical type n (%)**	Nodular Morphoeic Superficial	331 (89.2) 22 (5.9) 18 (4.9)
**High or low risk of clinical type n (%)**	Low risk High risk	331 (89.2) 40 (10.8)
**Pathologic type n (%)**	Nodular Infiltrative Micronodular Morphoeic Superficial Basosquamous Adenoid Keratotic	244 (65.8) 50 (13.5) 23 (6.2) 21 (5.7) 18 (4.8) 8 (2.1) 4 (1.1) 3 (0.8)
**High or low risk of pathologic type n (%)**	Low risk High risk	251 (67.7) 120 (32.3)
**Perineural invasion n (%)**	Negative Positive	(97.8) 8(2.2)

To perform accurate statistical analysis of the correlation between variables, site involvement was classified as nasal BCC and OSOF, also both pathological and clinical types were categorized as high- and low-risk types.

Based on the results, out of 328 patients with 371 BCC, 144 and 227 lesions were located on the nose and in OSOF, respectively. It was also found that the other common sites of involvement were in the periorbital area and forehead area with 57 (15.4%) and 30 (8.1%) cases, respectively. The involvement frequency of OSOF is presented in Table 1.The mean scores of patients' age, BCC size, and disease duration in the nasal area vs. 

OSOF were 53.79±9.657 vs. 57.92±7.949 years, 12.28 ±5.654 vs. 16.20±7.785 mm, and 37.83 ±9.856 vs. 44.39±8.687 months, respectively. There was a statistically significant difference between the study groups in terms of age, mean size, and duration (P<0.05) (Table.2).

**Table 2:** Correlation between involvement sites of basal cell carcinoma and variables

Variables		Nose	Other sites of face	P-value
**Gender n (%) **	Female Male	49 (36.6) 82 (63.4)	82 (40.5) 115 (59.5)	0.654
**Mean age**	Years	53.79±9.657	57.92±7.949	P<0.001
**Mean size**	mm	12.28±5.654	16.20±7.785	P<0.001
**Mean duration **	Months	37.83±9.856	44.39±8.687	P<0.001
**Personal history of basal cell carcinoma n (%)**	Yes N (%) No	54 (37.5) 90 (62.5)	66 (29.1) 161 (70.9)	0.091
**Number of lesions**	One Multiple	14 (10.7) 117 (89.3)	18 (9.1) 179 (90.9)	0.781
**Primary or recurrence lesion n (%)**	Primary Recurrence	99 (68.8) 45 (31.2)	182 (80.2) 45 (19.8)	0.013
**High or low risk of clinical type n (%)**	Low risk High risk	123 (85.4) 21 (14.6)	208 (91.5) 19 (8.5)	0.06
**High or low risk of pathologic type n (%)**	Low risk High risk	78 (54.2) 66 (45.8)	173 (76.2) 54 (23.8)	0.01
**Perineural invasion n (%)**	Negative Positive	139 (96.5) 5 (3.5)	224 (98.7) 3 (1.3)	0.275
				

It was revealed, among the 328 patients, 106 (32.3%) cases had a past medical history of BCC and 54 (37.5%) of patients with nasal BCC had a past medical history of BCC (P=0.091) (Tables 1,2). It was also found that 331 (89.2%), 22 (5.9%), and 18 (4.9%) lesions were nodular, morphoeic, and superficial clinical types, respectively. The high-risk clinical form was observed in 21(14.6%) and 19 (8.4%) lesions on the nose and OSOF, respectively (P=0.06) (Tables 1,2). 

The pathologic assessment of BCC revealed 244 (65.8%) nodular, 50 (13.5%) infiltrative, and 23 (6.2%) micronodular pathologic types. On the other hand, 251 (67.7%) and 120 (32.3%) BCC were reported to be as low- and high-risk pathologic types, respectively. Additionally, 66 (55%) and 54 (45%) cases of BCC on the nose and OSOF were reported as high-risk pathologic types, respectively (Tables 1,2). 

There was a significant difference between nasal BCC and OSOF in high-risk pathologic type of BCC (P=0.001) (Table.2). 

According to the results, among 371 BCC lesions, 281 (75.7%) and 90 (24.3%) presented as primary and recurrent BCC, respectively, of which 44 (48.9%) on the nose and 46 (51.1%) in OSOF had recurrent BCC.

 Perineural invasion was found in 8 (2.2%) patients with BCC, and 5 (3.5%) BCC -on the nose and 3 (1.1%) cases in OSOF had PNI (P=0.275) (Tables 1, 2).

## Discussion

The results of our study showed that patients with nasal BCC had significantly earlier onset, shorter duration, high-risk pathologic type, and smaller and recurrent lesions, compared to OSOF. In this study, most of the patients were male, without a personal history of BCC, mostly in their sixth decade of life, and with a mean of 41 months for the duration of the presentation. The BCC lesions had a mean size of 12 mm, presented mostly on the nose and were of primary, nodular, pathologic, and clinical types, and had rare PNI. 

Based on the results of the present study, the nose was found as the most common site of involvement, which was consistent with the findings of most previous studies (3,5,6-8). Although several factors are involved in the induction of BCC, sunlight has been found to have the most important etiologic role in the pathogenesis of BCC (1-3).

On the other hand, the nose is one of the sites that has the maximum exposure to sunlight; therefore, the nose is more susceptible to the development of BCC (11). The facial BCC is almost always observed among the middle-aged and elderly patients (1-8). Moreover, the results of a study (11) revealed that the mean age of patients with nasal BCC was 74.8 years, which seemed to be a little higher than that of OSOF. Conversely, in the current study, patients with BCC in OSOF were significantly older than those with nasal BCC. It is supposed that in the area of our study, the highly important etiologic role of sunlight and maximum exposure of the nose to sunlight resulted in the early onset of nasal BCC. Wollina et al. (11) showed nasal BCC was twofold more common in females than males; nevertheless, our male patients were slightly more involved with BCC on the nose than OSOF. This discrepancy in the findings may be related to the outdoor occupations and inappropriate sun protection in this area among males. Our patients uncommonly had multiple BCC on the nose and OSOF, which was consistent with the results of previous studies. Kiiski et al. (12) showed that multiple BCC is more common among young patients and those who had red hair and higher socioeconomic status. Nonetheless, most of our patients had brown skin and were in their sixth decade of life; consequently, there was no difference in terms of multiple BCC between the nose and OSOF. 

The findings of most studies (7,13,14) have shown that the majority of patients with facial BCC presented late, then the lesion grows, so large size is a risk factor. However, in our study, nasal BCC had an earlier presentation and smaller size than BCC in OSOF, which could be related to the cosmetic importance of the nasal unit, more exposure of BCC on the nose, and more recommendations of the patients’ companions to refer to medical centers. According to the results of various studies, nasal BCC is associated with a high rate of recurrence, which is consistent with our findings. The higher recurrence of BCC on the nose is attributed to its very high-risk location because of special anatomic situation, a fan-like extension of malignant cells, PNI, and underestimation of safe margin in primary lesions (7-10,15,16). Our findings indicated that nodular type was the most common low-risk pathologic type on both nose and OSOF, which was in line with the results of many studies (7,4,8,11). Nonetheless, high-risk pathologic type, especially infiltrative type, was significantly higher in the nose region. Consequently, a safer margin is necessary for the eradication of nasal BCC, especially in situations in which the intent is both to determine diagnosis and cure in one session. The results of some studies (7,11,13,17) were indicative of the slightly higher prevalence of the high-risk clinical type, including morphoeic form, on the nose than on the OSOF, which was in line with our findings. Therefore, a dermatosurgeon needs to be very careful in choosing a definitive treatment for nasal BCC. Since the perineural spread of BCC is associated with a more aggressive course and recurrence rate, the recognition of PNI is critical for the treatment of this malignancy (18,19). Although the prevalence of PNI in our patients was 2.2% and slightly higher in the high-risk pathologic type, nose location, and recurrent lesions, Santos et al. (19) found that PNI was significantly higher in large tumors and morphoeic pathologic type; however, no significant difference was observed in gender, age, and location. The researchers of the present study assumed that this difference might be related to the selection of cases, method of PNI identification, and expertise of the pathologist. Although this study was carried out in geographic areas with usual skin Fitzpatrick III and some different social behaviors, general scientific evaluation of patients was not an obstacle to the generalizability of its results, despite the mentioned factors. 

## Conclusion

Our findings showed that patients with nasal BCC presented significantly more recurrent lesions and more dangerous pathological types than OSOF. Moreover, nasal BCC had a slightly high-risk pathological type and PNI as an important risk factor. Therefore, considering the nasal site as an important cosmetic unit, the more limitation of the nose in performing any procedure, and the presence of more risk factors in the nasal area, the definite treatment of nasal BCC requires special attention, expertise, and experience.
